# Case of Urinary Concretion, Complicated with an Adipose Tumour of the Abdomen, Weighing Four Pounds and a Half, Avoirdupois

**Published:** 1817-01

**Authors:** Henry Ward

**Affiliations:** Member of the Royal College of Surgeons, London


### *Case of Urinary Concretion, complicated with an adipose Tumour of the Abdomen, weighing four pounds and a half, Avoirdupois.* By Henry Ward, Esq. Member of the Royal College of Surgeons, London.

On Monday morning, Sept. the 30th, 1816, I examined, in conjunction with Dr. Palmer, the body of Mr. D. a most respectable agriculturist, residing near Orton-on-the hill, in the county of Leicester. Forty-two hours had then elapsed from the time of death. Mr. D. had well-nigh attained his seventieth year. The following were the results of this highly interesting dissection.

*External view.* The face was much shrunk. The lower limbs were generally of a mottled, and, in several places, of a livid colour. The margin of the anus presented a dark purple appearance. The abdominal muscles were elevated by a large, firm, but elastic tumour; the limits of which may be thus defined: Inferiorly, it extended as far as Poupart's ligament; to the left, somewhat beyond the linea alba or mesial line: superiorly, about one inch and a half above the umbilicus; and, in a dextro lateral direction, it was in contact with the anterior spinous process, and cristal margin of the right ilium. The situation of the tumour bespoke that it was not formed by the distended bladder. A more probable conjecture was, that we should find it consisting of an enormously enlarged kidney.*

*Internal appearances. Abdomen:* There was an uncommon accumulation of fat in the cellular structure of the integuments of the abdomen. Upon exposing the cavity, in the ordinary manner, by a crucial incision, the sheet of peritonæum, lining the abdominal muscles, was found thickened, and strongly adherent to the tumour of the right side. This, when fairly uncovered, was seen pushing upwards, and to the left, the small intestines. The caput coli, with a small portion of the ascending colon, was intimately connected with the front of the tumour at its superior part. This latter, in fact, was seated between the *psoas, iliacus internus, and quadratus lumborum muscles* of the right side, and the peritonæum, which, naturally covering them, had

---

* This conclusion was rendered at least plausible by reflecting on the singular and interesting case of abdominal disease, so ably detailed by Dr. Johnson in the commencement of the second volume of the *Medico-Chirurgical Journal and Review*.

been separated by the encroachment of the tumour. The portion of peritonæum, thus partially investing the mass, and closely adhering to its whole anterior surface, was both thickened and inflamed. The right ureter took its course along the left boundary of the tumour, and thus crossed the vertebral column to reach the pelvis. The trunks of the inferior vena cava and abdominal aorta were thrust by it considerably to the left, as was also the urinary bladder. The left ureter, much enlarged, deviated but little from its wonted route. For the sake of obtaining a clearer view of the tumour, it was found necessary to remove the intestinal canal, from the commencement of the jejunum to that of the rectum. The enormous mass, now fully exposed, conveyed to the finger a sense of fluctuation; but, upon incision, it was found to consist entirely of adipose substance. Much difficulty was encountered in separating it from its connections with the *psoas* and *iliacus internus muscles*, and from the external iliac artery and vein, and the anterior crural nerve, to which also it had contracted an adhesion. This done, it was found descending below the inferior border of the external oblique muscle (Poupart's ligament) and firmly bound down by it. Hence, it became necessary to divide freely the crural arch and femoral fascia. By these means, the origin of the tumour was, at length, traced to the upper part of that triangular space in the thigh; the base of which is formed by Poupart's ligament, and the sides by the *Sartorius* and *triceps adductor muscles.* The body of the tumour itself was divided into two distinct and unequal portions; or rather, it consisted of two tumours united by a narrow isthmus. The smaller, and inferior of these, about the size of a large orange, was situated below Poupart's ligament, and bound down by the fascia of the thigh: the superior, possessing the volume of a child's head, rose above the crural arch, and occupied the site behind the peritonæum, previously described. The deep sulcus, separating the two portions, corresponded to the situation of Poupart's ligament.

The whole tumour, on removal, weighed four pounds and a half.* The urinary bladder was contracted upon a

---

* For my accuracy in this respect, I might, if it were necessary, appeal to Dr. Pigot, of Chester, who that day called at my house, and saw the tumour weighed. He mentioned having, some years since, seen a pendulous tumour, of somewhat similar structure, removed from the perinæum of the human subject, by Mr. Hutchinson, the learned author of large, egg-shaped, rugged concretion, apparently consisting of oxalate of lime, and weighing two ounces six drachms. The prostate gland was in an enlarged and evidently morbid condition. The pelvis of the left kidney contained a quantity of purulent fluid; and every trace of the original structure of the organ was obliterated. The same process of morbid alteration was visibly beginning in the right kidney. The intestinal canal, spleen, pancreas, and substance of the liver, were healthy; but, in the gall-bladder, there were one large, and numerous small biliary calculi, firmly adherent to its internal coat. They consisted of a brightish, gold-coloured, crystalline substance.† The canal of the abdominal aorta was much contracted at the point where it sustained the pressure of the tumour; and ossification had commenced in the internal coat of the vessel, about the origin of the inferior mesenteric artery. The viscera of the *thorax* were perfectly sound. No symptom had arisen during life to denote the existence of any lesion within the *cranium;* and hence I would not needlessly encroach upon the liberality of Mr. D——'s family, in requesting permission to examine that cavity.

*History.* Mr. D—— had been a stout man, of active and temperate habits, and constantly engaged in agricultural pursuits. In fact, he possessed all that vigour of body, all those generous and estimable qualities of the mind, which so conspicuously distinguish the character of the English farmer. Five years ago, the symptoms of disease in the urinary organs were first declared. Till that period, he had enjoyed uninterrupted health. The ordinary means were employed for his relief, with variable and transient success. About the close of August, 1815, he was visited by Dr. Palmer, who confirmed the opinion of his surgeon, Mr. Whitby, of this place, as to the existence of a stone in the bladder. At this time, enlargement and tenderness of the prostate gland were ascertained by examination from the rectum; but the irritable and obstructed state of the urethra utterly precluded the introduction of the sound. Dr. Palmer assures me, that no tumour of the abdomen was then visible. The warm bath the *Biographia Medica.* An account of it may be seen in one of the earlier volumes of that once valuable, but now degenerate work, *The Medical and Physical Journal.*

† See the extraordinary cases of hydrocele, *Med. Chirurg. Journal and Review,* vol. ii. page 79.

and alkalies were prescribed with temporary relief; and Mr. D. continued to walk about his grounds till within ten days of his decease. But, for some time, he had been observed to incline towards the *right* side, as well in the sitting as in the erect posture; and was heard to complain of a sense of pain and weakness in the corresponding hip. On the 21st of September, I first visited Mr. D. during the illness of his regular medical attendant. He was then labouring under the symptoms of stone in their most violent form. Upon examining the abdomen, my attention was strongly arrested by the tumour, which constitutes the principal feature of the case; but I felt quite at a loss to explain its nature or origin. The *left* inferior extremity was almost destitute of sensation, œdematous, cold up to the knee, and presented a livid appearance. Without any marked alteration in his symptoms, Mr. D. continued gradually to decline till the evening of the 28th, when he expired.

In conclusion, it may be remarked, as an extraordinary circumstance, that a tumour, of the volume described, should have been formed in the short space of one year. I consider it highly probable, that this tumour was totally unconnected with the affection of the urinary organs; that it began in morbid alteration of one of the inguinal glands; and that, restricted in its growth outwards, by the strong fascia of the thigh, it had gradually made way where less resistance was opposed to its progress, risen behind Poupart's ligament, and insinuated itself between the peritonæum and the iliac and lumbar muscles. Any surgical operation for the removal of a tumour thus situated and connected, even if its precise nature could, by any means, have been distinguished during the life of the patient, seems to be completely out of the question.

*Atherstone, November* 1816.

